# A Case of Injury of the Spine—Enormous Accumulation of Intestinal Gas—Evacuation with Aspiration—Recovery

**Published:** 1876-10

**Authors:** E. W. Lee

**Affiliations:** Chicago


### A CASE OF INJURY OF THE SPINE—ENORMOUS ACCUMULATION OF INTESTINAL GAS—EVACUATION WITH ASPIRATION—RECOVERY.

By E. W. LEE, M.D., Chicago.

On the evening of the ninth of May I was called to visit J. K., aged thirty-five years, and obtained from him the following history of his case:

Four days previously he was driving his team under a shed, and the wagon-seat being higher than that on which he was accustomed to ride, he got caught between the seat and roof, and was severely crushed. He was removed to his home and a physician called to attend him. His back had been assiduously rubbed with liniments, etc. There had been no action of the bowels from the time of the injury, notwithstanding that purgatives had been freely administered, also enemata. Having patiently listened to his story, I proceeded to examine him, and first feeling his pulse, I was very much astonished to find it extremely feeble—in fact so easily obliterated as to make it a very difficult matter to count it. As well as I could make out, it was not over 115 to 120; his skin was cold, and he was bathed in profuse perspiration; the countenance had that sunken, pinched appearance characteristic of collapse—*his voice was strong*—the whole abdomen was enormously distended. He had been vomiting continuously, the discharges having the odor, as he expressed it, "of a Bohemian water closet." I saw at once that it would be useless to go through the form of administering purgatives, enemata, etc., besides they had been thoroughly tried. I injected $\frac{1}{8}$ gr. sulph. morph. hypodermically to begin with, and as the case seemed desperate, thought the best chance to save the man's life was to directly liberate the accumulation of gas in the abdomen.

I provided myself with an aspirator, and with counsel in the person of my friend Dr. Cooley, who entirely coincided with me as to the course of treatment to be pursued, I selected the smallest needle of the aspirator, and boldly plunged it to the hilt into the most prominent point on the left side; a quantity of gas escaped, making a noise not unlike a "Kidder" battery in operation; this gas had a most sickening smell, and we judged from it that sphacelus had already set in; this impression being made stronger by the fact that after we had inserted the needle (larger size) into the right side and into the stomach, each time liberating a quantity of gas, and sensibly diminishing the distension, and the morphine having had ample time to have its full effect; his pulse was still weaker, almost gone; his skin colder, and the perspiration more profuse. We supposed the man would not live more than three or four hours (this was at 9 P. M.).

Next morning I was again sent for, and to my surprise heard that the bowels had spontaneously acted three or four hours after we left, and simultaneously the vomiting had ceased; the pulse had returned at the wrist; the distension was considerably diminished; he had partaken of and retained some nourishment, and felt much better. I turned him on his side in order to examine his back. The whole surface was contused, and about the lower end of the dorsal region I discovered that a couple of the spines of the vertebra were fractured. The man went on very well from that time, and has made so far a good recovery. The only hopeful symptom about this case was that the pulse was not high, at the most I do not think it reached 120, but it was impossible to get an accurate count.

I publish this case because I have no doubt that the liberation of the gas proved a strong stimulant to the restoration of peristaltic action, and also as illustrating the impunity with which the peritoneum may be punctured.

